# *simplifyEnrichment*: A Bioconductor Package for Clustering and Visualizing Functional Enrichment Results

**DOI:** 10.1016/j.gpb.2022.04.008

**Published:** 2022-06-06

**Authors:** Zuguang Gu, Daniel Hübschmann

**Affiliations:** 1 Molecular Precision Oncology Program, National Center for Tumor Diseases (NCT) Heidelberg, D-69120 Heidelberg, Germany; 2 Heidelberg Institute of Stem Cell Technology and Experimental Medicine (HI-STEM), D-69120 Heidelberg, Germany; 3 German Cancer Consortium (DKTK), D-69120 Heidelberg, Germany; 4 Department of Pediatric Immunology, Hematology and Oncology, University Hospital Heidelberg, D-69120 Heidelberg, Germany

**Keywords:** Functional enrichment, Simplify enrichment, Clustering, R/Bioconductor, Software, Visualization

## Abstract

**Functional enrichment** analysis or gene set enrichment analysis is a basic bioinformatics method that evaluates the biological importance of a list of genes of interest. However, it may produce a long list of significant terms with highly redundant information that is difficult to summarize. Current tools to **simplify enrichment** results by **clustering** them into groups either still produce redundancy between clusters or do not retain consistent term similarities within clusters. We propose a new method named *binary cut* for clustering similarity matrices of functional terms. Through comprehensive benchmarks on both simulated and real-world datasets, we demonstrated that *binary cut* could efficiently cluster functional terms into groups where terms showed consistent similarities within groups and were mutually exclusive between groups. We compared *binary cut* clustering on the similarity matrices obtained from different similarity measures and found that semantic similarity worked well with *binary cut*, while similarity matrices based on gene overlap showed less consistent patterns. We implemented the *binary cut* algorithm in the R package *simplifyEnrichment*, which additionally provides functionalities for visualizing, summarizing, and comparing the clustering. The *simplifyEnrichment* package and the documentation are available at https://bioconductor.org/packages/simplifyEnrichment/.

## Introduction

Functional enrichment analysis or gene set enrichment analysis is a method widely used to evaluate whether genes of interest (GOIs), *e.g.,* differentially expressed genes, are over-represented or depleted in certain biological processes represented by pre-defined gene sets. It is performed by testing the list of GOIs against these gene sets through a hypergeometric test [1], by analyzing whether the gene sets are enriched at the top of a ranked gene list [2], or by evaluating the gene expression profile in individual gene sets through univariate or multivariate approaches [3]. There are also methods to associate genomic regions of interest (ROIs), *e.g.*, regulatory domains, to gene sets to identify which biological processes the ROIs might affect [4]. Functional enrichment analysis depends on *a priori* biological knowledge encoded in pre-defined gene sets, which are obtained from multiple sources. Among them, Gene Ontology (GO) [5] is the most comprehensive source, which organizes the biological terms in a hierarchical tree structure in the form of a directed acyclic graph (DAG). Other popular sources are, for example, Kyoto Encyclopedia of Genes and Genomes (KEGG) pathways [6] and Molecular Signatures Database (MSigDB) [7]. Depending on the context, gene sets are also referred to as functional terms, and we follow that convention in this paper.

Enrichment results usually contain a long list of enriched terms that have highly redundant information and are difficult to summarize. For example, an analysis of 671 EMBL-EBI Expression Atlas differential expression datasets [8] using GO gene sets with the biological process (BP) ontology showed that there were 543 (80.9%) datasets having more than 250 significantly enriched GO terms under false discovery rate (FDR) <0.05. The enrichment results can be simplified by clustering functional terms into groups where in the same group, the terms provide similar information. The similarities between terms are important for clustering. The calculation varies for different sources of gene sets. In the simplest and most general form, the similarity between two gene sets is based on gene overlap, *i.e.*, the presence or absence of genes in gene sets. It is calculated as the Jaccard coefficient, Dice coefficient, or overlap coefficient [9]. The kappa coefficient is suggested to be more robust, and it is implemented in the widely used tool, Database for Annotation, Visualization and Integrated Discovery (DAVID) [10]. For GO gene sets, there are more advanced methods that integrate the DAG structure to measure the semantic similarity between terms, *e.g.,* the methods proposed in [11], [12] and implemented in the R package *GOSemSim* [13]. A comprehensive review of semantic measures of GO terms can be found in [14]. Semantic similarity can also be calculated on other ontologies with DAG structure, such as the Disease Ontology (DO) [15], [16].

There are methods that reduce the number of terms by manually selecting a small subset, such as GO slims [17], or by selecting the most significant terms [18] or the most representative terms [19], [20]. However, ignoring the rest of the terms causes a loss of information. A more advanced way to simplify the enrichment results is to cluster the similarity matrix of gene sets. Various clustering methods that have already been implemented in current tools can be applied, for example, hierarchical clustering [20], affinity propagation clustering [21], or density-based clustering [10]. The similarity matrix can also be treated as an adjacency matrix and converted to a graph where the gene sets are the nodes, and the similarity values are the weights of edges. Then, algorithms for detecting graph communities or modules can be applied to identify the clusters. A popular tool is Enrichment Map [9].

Nevertheless, there are some common issues with clustering the similarity matrix of gene sets. Taking GO gene sets as an example, we call the root nodes (*i.e.*, the three top terms of BP, molecular function, and cellular components) *the roots* of the GO tree. In the GO tree, there are branches with different depths, which causes two major problems for clustering. First, the size of GO clusters varies a lot, which means that there are large clusters that normally correspond to large branches inheriting from the root region of the GO tree, while at the same time, there are also many tiny clusters that correspond to the small branches in the very downstream part of the GO tree. Due to the hierarchical relations in the tree, large clusters tend to have relatively low average similarities among terms, while small clusters generally have relatively high average similarities. Methods such as *k*-means clustering cannot preserve the large clusters and instead split them into smaller clusters, which might still produce redundant information between clusters. Moreover, they are not able to separate tiny clusters and prefer to merge them into one larger cluster. These over-segmentation and under-segmentation behaviors are mainly due to the fact that *k*-means clustering expects clusters to have similar sizes. Second, large GO clusters contain large amounts of terms, and it is possible that a small fraction of terms in cluster A also shares similarities to terms in cluster B, *i.e.*, larger clusters are less mutually exclusive than smaller clusters, and this results in some graph community methods merging clusters A and B into one larger cluster due to the existence, albeit in small amounts, of terms shared by the two clusters. This, in turn, increases the inconsistency among gene sets in that merged cluster. Thus, an effective method is needed to balance these two scenarios, reflecting the generality and specificity of the GO system.

In this study, we propose a new method named *binary cut* that clusters functional terms based on their similarity matrix. It recursively applies partitioning around medoids (PAM) with two groups on the similarity matrix, and in each iteration step, a score is assigned to the submatrices to decide whether the corresponding terms should be further split into smaller clusters or not. We compared *binary cut* to a wide range of other clustering methods with 100 simulated GO lists and 485 GO enrichment results from real-world datasets, and we demonstrated that, with similarities calculated by semantic measurements, the *binary cut* was more efficient to cluster GO terms such that inside clusters, terms shared more consistent similarity, while at the same time, the clusters had a higher degree of mutual exclusivity. *Binary cut* can also be applied to similarity matrices in general, *i.e.*, based on gene overlap between gene sets of any type. However, the performance of *binary cut* varied, depending on the sources of gene sets. Similarity matrices based on gene overlap generally showed less consistent patterns than semantic similarity matrices and were not suggested to be used with the *binary cut*.

We implemented the *binary cut* algorithm in a R/Bioconductor package named *simplifyEnrichment*. After the functional terms have been clustered, *simplifyEnrichment* visualizes the summaries of clusters by word clouds, which helps users easily interpret the common biological functions shared in the clusters. *simplifyEnrichment* can also export the clustering results to a web application so that users can interactively retrieve information on functional terms that belong to a certain cluster. Additionally, *simplifyEnrichment* provides a framework where user-defined clustering methods can be integrated, and subsequently, their results can be fed into the visualization functionality of *simplifyEnrichment*. Furthermore, *simplifyEnrichment* can compare multiple results from different clustering methods in a straightforward way.

## Method

### Similarity measure

If functional terms can be clustered into groups, the similarity matrices of corresponding gene sets are expected to represent a pattern where blocks can be observed on the diagonal, where hierarchical clustering is applied on both rows and columns. Thus, efficiently clustering gene sets is equivalent to the task of identifying the *diagonal blocks* in the similarity matrix. The similarity between gene sets can be calculated as gene overlaps by, *e.g.*, Jaccard coefficient, Dice coefficient, or overlap coefficient. The three coefficients have similar forms. It has also been suggested to calculate the gene overlap by kappa coefficient, which takes into account the possibility of gene occurrence in two gene sets just by chance [10]. The formal definition of the four gene overlap coefficients can be found in File S1. If the ontologies have a DAG structure such as GO, the topology information can be used to calculate the similarities, to generate a so-called semantic similarity, *e.g.*, through information content (IC)-based approaches which measure the information each term obtains from its offspring terms. There exist a broad number of algorithms for calculating semantic similarities; see [14] for a comprehensive overview.

As we observed from the benchmark datasets for GO terms, semantic similarities showed more distinct diagonal block patterns than similarity measures based on gene overlap (see comparisons in the section “general similarity by gene overlap”). The diagonal block pattern of the semantic matrix was frequently observed even for randomly sampled GO terms (Files S2 and S3). Thus, we mainly use semantic similarity for clustering GO terms in this work. *simplifyEnrichment* uses the semantic measurements implemented in the R package *GOSemSim* [13] and takes the relevance method [12] as default (see comparisons of different semantic measurements in File S4). Nevertheless, a similarity matrix by any other method and on any other ontology can be provided for analysis with *simplifyEnrichment*.

### Clustering process

**Figure 1:**
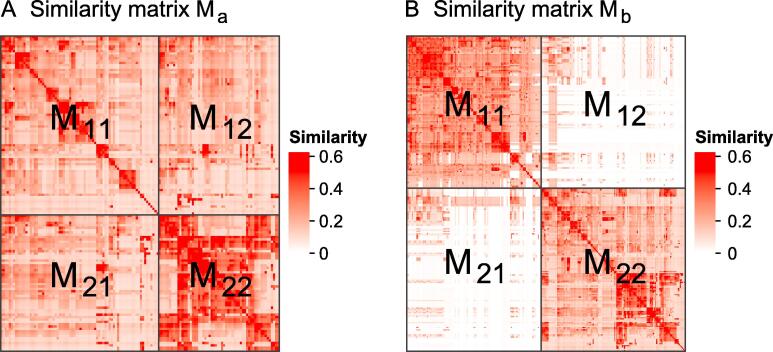
**Examples of similarity matrices for two sets of GO terms** The two matrices are denoted as M*_a_* and M*_b_*. Both matrices are split into two groups in the two dimensions, where submatrices are labeled as M_11_, M_12_, M_21_, and M_22_. GO, Gene Ontology.

Let M*_a_* and M*_b_* denote the similarity matrices of two lists of GO terms, as illustrated in Figure 1. Intuitively in this example, GO terms in M*_a_* have overall high pairwise similarities; thus, they should be treated as one single cluster (Figure 1A), while terms in M*_b_* show a two-block pattern on the diagonal and should be split into two sub-clusters (Figure 1B). A metric can be designed to decide whether a group of terms should be split or not. Based on this idea, we propose a new method named *binary cut* to cluster the similarity matrix of functional terms, executed in two phases:

*Phase 1: applying divisive clustering and generating a dendrogram*

This phase includes two steps. In the first step, for the similarity matrix M of a given list of GO terms, applying a certain partitioning method (*e.g.*, PAM) with two-group classification on both rows and columns. It partitions M into four submatrices, denoted as M_11_, M_12_, M_21_, and M_22_, where the indices represent the groups in the two matrix dimensions. Next, calculating the following scores *s*_11_, *s*_12_, *s*_21_, and *s*_22_ for the four submatrices. Taking *s*_11_ as an example, let *X* denote the vector of entries in M_11_, and *F_X_*(*x*) the cumulative distribution function (CDF) of *X*. Then *s*_11_ is calculated as in Equation (1).

(1) $$s_{11}=1-\int_{0}^{1}F_{X}\left(x\right)dx$$

Please note, when calculating *s*_11_ and *s*_22_, entries on the diagonal of M_11_ and M_22_ are excluded. Since the similarity matrix is always symmetric, *s*_12_ and *s*_21_ are equal. *s*_11_ or *s*_22_ is defined to be 1 if M_11_ or M_22_ have only one row. We then define the score *s* as in Equation (2).

(2) $$s=\frac{s_{11}+s_{22}}{s_{11}+s_{12}+s_{21}+s_{22}}$$

The diagonal blocks M_11_ and M_22_ correspond to similarity measures within sub-clusters, whereas the off-diagonal blocks M_12_ and M_21_ correspond to similarity measures between sub-clusters. In a successful clustering, similarity measures will be higher within clusters than between clusters, and this roughly results in *s*_11_ + *s*_22_ ≥ *s*_12_ + *s*_21_. Thus, the values of *s* approximately range between 0.5 and 1 (The probability of *s* smaller than 0.5 is almost 0 with random GO datasets and 0.0003 with the Expression Atlas datasets. See details in File S5). If M shows overall similarities, *s* is far less than 1 (Figure 1A), and it should not be split anymore, while if the GO terms can still be split into more groups, *s*_12_ and *s*_21_ are close to 0, which results in *s* close to 1 (Figure 1B).

In the second step, apply the first step to the two submatrices, M_11_ and M_22,_ respectively. The clustering in the two steps is executed recursively, and the process is saved as a dendrogram where the score *s* is attached to every node in the dendrogram, which corresponds to every submatrix in the iteration. The clustering stops when the number of terms in a group reaches 1; these are the leaves of the dendrogram.

*Phase 2: cutting the dendrogram and generating clusters*

Since every node in the dendrogram has a score *s* computed in Phase 1, *s* is simply compared to a cutoff with 0.85 as the default. *simplifyEnrichment* provides functions that help to decide an optimized cutoff value by testing the performance of clustering with a list of cutoffs. If *s* is larger than the cutoff, the two branches from the node are split, else, all the terms under the node are taken as a single cluster.

Nodes with large numbers of terms tend to have relatively smaller *s*; thus, it is possible that at a certain node, *s* does not exceed the cutoff but is very close to it, while its child nodes have values of *s* larger than the cutoff. In this case, we don’t want to close the node so early, and we still split this node into two subgroups so that its child nodes can be split furthermore. Thus, the rule in Phase 2 is modified as follows: if the score *s* of a given node does not exceed the cutoff but it is larger than 0.8× cutoff, the node is still split if at least one of its child nodes has a score that exceeds the cutoff. This is equivalent to reassigning the maximal scores of its two child nodes to the score *s* of this given node. Note this scenario does not occur often, and the value of 0.8 is empirically determined based on a larger number of real-world datasets.

**Figure 2:**
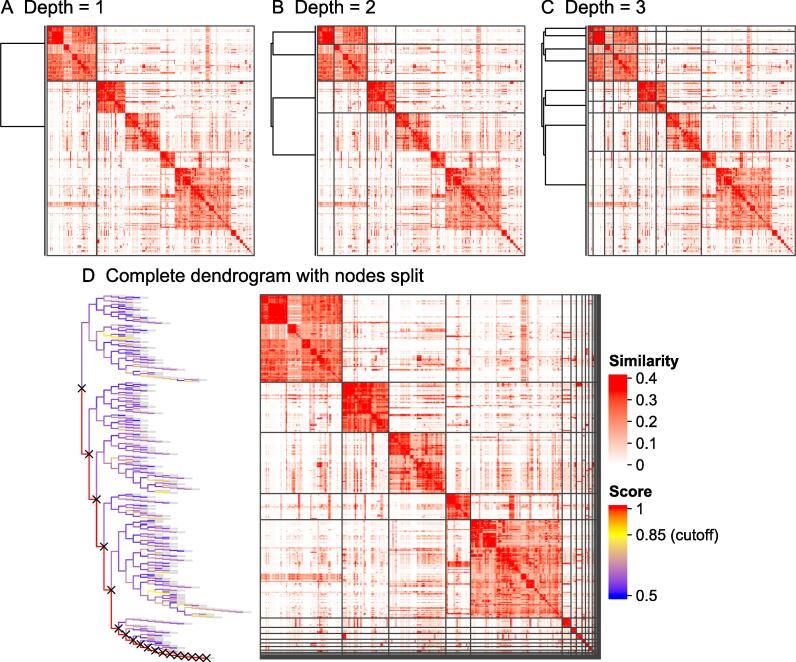
**A demonstration of the *binary cut* clustering with 500 random GO terms** **A.** The clustering in the first iteration. **B.** The clustering in the second iteration. **C.** The clustering in the third iteration. **D.** The complete dendrogram from *binary cut* clustering. The colors of the dendrogram segments correspond to the scores *s* assigned to the nodes. Nodes to split are marked with crosses.

An example of the process of *binary cut* clustering is demonstrated in Figure 2. Figure 2A–C illustrates the clustering in the first three iterations. Figure 2D illustrates the final dendrogram where the nodes that are split are marked with crosses. To optimize the clustering process, *simplifyEnrichment* supports partial clustering to reduce the computing time where the complete dendrogram is not generated, and the bifurcation stops on a node as long as the corresponding *s* does not exceed the cutoff.

### Software implementation

The *binary cut* algorithm is implemented in a R/Bioconductor package named *simplifyEnrichment*. The input of the package is a similarity matrix that is provided either directly by the user or by the functions implemented in *simplifyEnrichment*, such as the function *GO_similarity()* for calculating GO semantic similarity or the function *term_similarity()* that measures general gene set similarity by Jaccard coefficient, Dice coefficient, overlap coefficient or kappa coefficient based on gene overlap.

**Figure 3:**
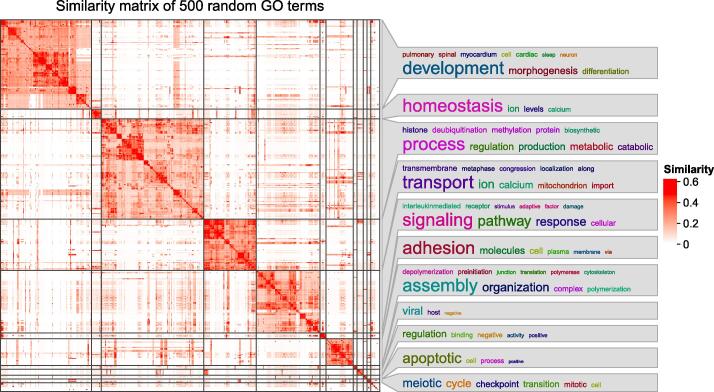
**Example of a similarity heatmap from 500 random GO terms that have been clustered and annotated with word clouds** The bottom right cluster with no word cloud annotation contains all other small clusters with numbers of terms less than 5. The plot was made by the function *simplifyGO()*.

The function *simplifyGO()* performs clustering for GO terms and visualizes the results. Once the GO terms have been clustered, the biological descriptions for the terms are automatically extracted. The summaries of the biological functions in clusters are visualized as word clouds and are attached to the similarity heatmap, which gives a direct iallustration of the common biological functions involved in each cluster (Figure 3). In word clouds, enrichment of keywords is assessed by Fisher’s exact test, and the significance is mapped to the font size of keywords. The function *simplifyEnrichment()* performs clustering on similarity matrices from any type of ontology. The static heatmap demonstrated in Figure 3 can be exported to a Shiny web application by the function *export_to_shiny_app()* so that users can interactively select GO terms that belong to a specific cluster from the heatmap for deeper exploration (File S6).

*simplifyEnrichment* also allows the integration of other clustering methods. New clustering functions can be added by the function *register_clustering_methods()*. The function *compare_clustering_methods()* applies multiple clustering methods on the same similarity matrix and compares them via heatmaps.

## Results

We compared the following 10 clustering methods to *binary cut*: *k*-means clustering (*kmeans*), where the optimized number of clusters was selected based on the distribution of the within-cluster sum of squares (WSS), PAM where the optimized number of clusters was selected by the R package *fpc* (https://CRAN.R-project.org/package=fpc), dynamicTreeCut [22], mclust [23], apcluster [24], *hdbscan* [25], the following graph community methods implemented in the R package *igraph* [26]: fast greedy, louvain and walktrap, as well as one additional community method MCL [27] which was recently used in implementation for clustering GO terms [28]. These selected methods are based on various categories of clustering algorithms and are by default supported in *simplifyEnrichment*. They were applied with their default clustering parameters in the benchmark. GO semantic similarities were calculated by the relevance method [12] with the R package *GOSemSim*.

### Application of *simplifyEnrichment* to random GO lists

**Figure 4:**
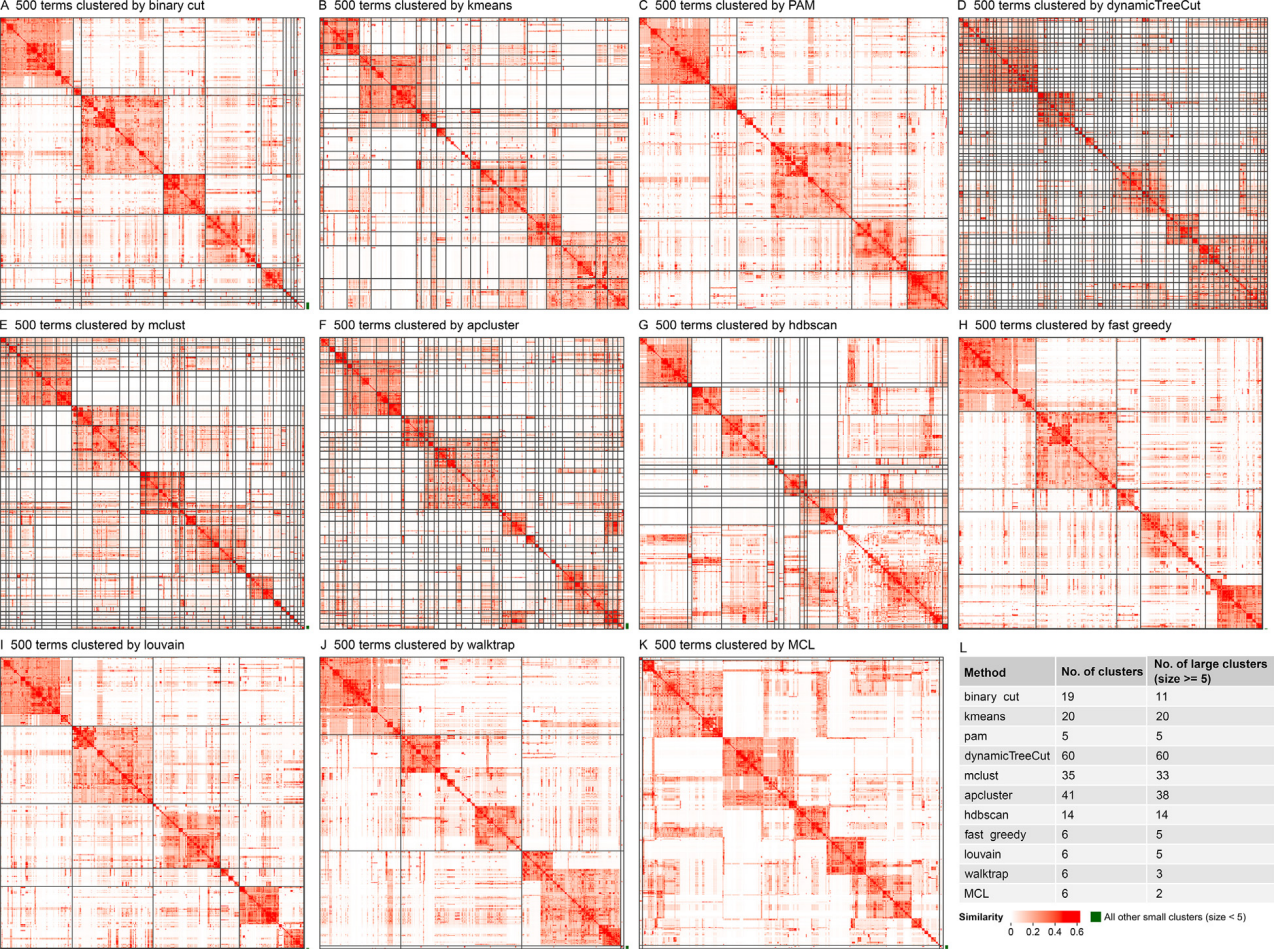
**Comparison of different clustering methods** **A.** Clustering by *binary cut*. **B.** Clustering by *kmeans*. **C.** Clustering by PAM. **D.** Clustering by dynamicTreeCut. **E.** Clustering by mclust. **F.** Clustering by apcluster. **G.** Clustering by *hdbscan*. **H.** Clustering by the fast greedy graph community method. **I.** Clustering by the louvain graph community method. **J.** Clustering by the walktrap graph community method. **K.** Clustering by the MCL graph community method. **L.** Numbers of all clusters and numbers of large clusters with size ≥ 5. For some methods, the small clusters (size < 5) were put into one single cluster on the bottom right of the heatmap and were marked by green lines. All the methods were applied to the same GO semantic similarity matrix from 500 random GO terms from the biological process ontology. The plots were generated by the function *compare_clustering_methods()*. PAM, partitioning around medoids.

500 random GO terms were uniformly sampled from the BP ontology. Clusterings of 11 different methods are illustrated in Figure 4. dynamicTreeCut, mclust, and apcluster generated huge numbers of small clusters (Figure 4D–F). *Kmeans* and *hdbscan* generated intermediate numbers of clusters, but *kmeans* failed to preserve large clusters even if overall similarities within them were high (*e.g.*, the first three clusters in Figure 4B), while for *hdbscan*, the largest cluster did not show consistent similarities for all term pairs and it should be split further (the bottom right cluster in Figure 4G). PAM as well as community methods of fast greedy, louvain, walktrap and MCL identified large clusters; however, some large clusters may still be split further (Figure 4C and H–K), as most strikingly visualized for MCL where only one large cluster was identified under its default clustering parameters (Figure 4K). In comparison, *binary cut* generated clean clusters and it was able to identify large and small clusters at the same time (Figure 4A).

We then benchmarked the clustering methods quantitatively. To this end, the random GO lists of 500 BP terms were generated 100 times, and the following metrics were used:

*Difference score*

It measures the difference in the similarity values of the terms within clusters and between clusters. For a similarity matrix denoted as M, and for terms *i* and *j* where *i* ≠ *j*, the similarity value *x_i_*_,_ *_j_* is saved to the vector x_1_ when terms *i* and *j* are in the same cluster. *x_i_*_,_ *_j_* is saved to the vector x_2_ when terms *i* and *j* are in different clusters. The difference score measures the distribution difference between x_1_ and x_2_, calculated as the Kolmogorov-Smirnov statistic between the two distributions of x_1_ and x_2_.

*Number of clusters*

For each clustering, there are two numbers: the total number of clusters and the number of clusters with size ≥ 5 (*i.e.*, the large clusters). The two values can be used to test whether the clustering methods can identify small clusters.

*Block mean*

It is calculated as the mean value of the diagonal blocks in the similarity heatmap. It measures the average within-cluster similarity of the clustering. Using the same convention as for the difference score, the block mean is the mean value of x_1_. As demonstrated in Figure 4, when GO terms are over-clustered, clusters would have a high average within-cluster similarity, while when GO terms are under-clustered, the average within-cluster similarity tends to be low. Thus, the block mean value measures the balance between over-clustering and under-clustering. An intermediate value is representative of good classification.

*Comparison of clustering results*

**Figure 5:**
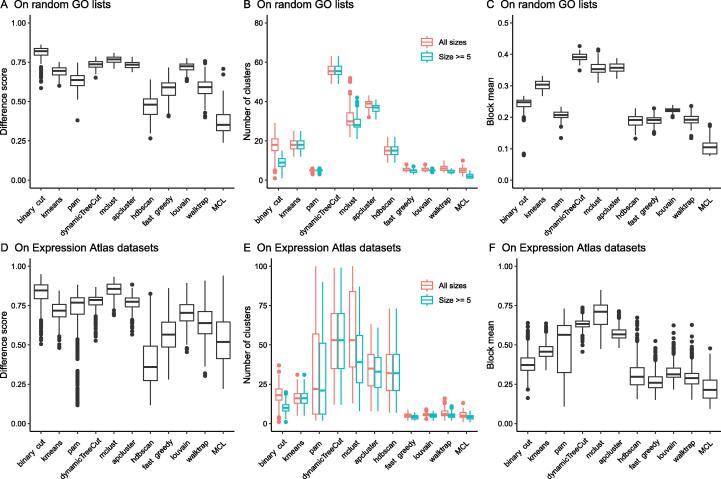
**Benchmarks of different clustering methods** **A.** Difference scores. **B.** Numbers of clusters. **C.** Block mean values. The analysis in A−C was applied to 100 random GO lists of 500 BP terms. **D.** Difference scores. **E.** Numbers of clusters. **F.** Block mean values. The analyses in E and F were applied to the functional enrichment results from 485 Expression Atlas datasets. BP, Biological Process.

As shown in Figure 5A, the *binary cut* had the highest difference score, reflecting that clusterings obtained with *binary cut* had the most distinct differences in similarity values within clusters and between clusters, therefore, the clusters identified by *binary cut* were the most mutually exclusive among all methods. dynamicTreeCut and apcluster generated a huge number of clusters (Figures 4D, F and 5B). These two methods can be considered too stringent for clustering GO terms; in addition, there still are high levels of redundant information among different clusters due to high between-cluster similarities. In comparison, graph community methods and *binary cut* generated moderate numbers of clusters, and especially for *binary cut*, the numbers of “large clusters” (size ≥ 5) dropped dramatically compared to the total numbers of clusters (Figure 5B), indicating that *binary cut* was able to identify small clusters. *Binary cut* generated clusters with moderate block mean values (Figure 5C). In comparison, *kmeans*, dynamicTreeCut, mclust, and apcluster generated high block mean values, reflecting that they were not able to preserve large clusters with intermediate similarities. Graph community methods generated low block mean values, mainly due to the fact that terms in the clusters did not necessarily need to have high similarities to all other terms, as long as terms in the graph community had enough connections (Figure 4H–K). The method *hdbscan* generated intermediate numbers of clusters and had intermediate block mean values, but it had the second-lowest difference score in the comparisons, which implies that differences between similarities within clusters and between clusters were small (Figure 4G). In conclusion, these comparisons indicated that *binary cut* kept both generality and specificity of GO clusters. The analysis reports for all 100 random GO lists can be found in File S3.

Of note, the *similarity* matrices of GO terms based on semantic measures showed clear *diagonal block* patterns even for lists of random GO terms. In File S4, we compared the five semantic similarity measurements supported in the *GOSemSim* package and found that the methods “Rel” (*i.e.*, the relevance method) [12], “Resnik” [29] and “Lin” [30] produced similar and clear *diagonal block* patterns. They are thus suitable for use with *binary cut* clustering. Furthermore, an analysis of the complete set of terms of GO BP ontology showed that with semantic similarity, globally, the similarity matrix had *diagonal block* patterns where the clusters corresponded to several top functional categories (File S7). Uniformly sampling from all BP terms tends to retain these modular patterns.

By default and in every iteration step, *simplifyEnrichment* partitions the current submatrix into two groups using PAM. In File S8, we compared *binary cut* clustering on 100 random GO BP lists with the following three partitioning methods: PAM, *k*-means++, and hierarchical clustering with the “ward.D2” method. *k*-means++ is an optimized *k*-means clustering by pre-selecting proper initial centroids [31], [32]. The results showed that the three partitioning methods performed very similarly, *i.e.*, with similar difference scores, numbers of clusters, and block mean values among 100 random GO lists. The final clusterings of GO terms agreed very well (on average 85.2% agreement, File S8) between the three partitioning methods. The three methods are all supported in *simplifyEnrichment*, and they can be selected specifically for individual datasets. *simplifyEnrichment* also supports running all three methods simultaneously for analysis and automatically selecting the method that generates clustering with the highest difference score.

### Application of *simplifyEnrichment* to real-world datasets

To assess the performance of *simplifyEnrichment* also on actual biological data, we analyzed all datasets from the Expression Atlas [8] (https://www.ebi.ac.uk/gxa/download) that have differential expression analysis results. We applied functional enrichment analysis with the R package *clusterProfiler* [18] to the significantly expressed genes (FDR < 0.05) with the GO BP ontology. We only took those datasets for which the number of significant genes was in the interval [500, 3000] and the number of significant GO terms (FDR < 0.05) was in [100, 1000]. This yielded 485 GO lists. Figure 5D–F illustrates the results of the comparisons among the 11 clustering methods. The conclusion was very similar to the benchmark with random GO datasets: *binary cut* outperformed other clustering methods. The analysis reports for 485 individual Expression Atlas datasets can be found in File S3.

We mainly benchmarked GO term enrichment with the BP ontology, which is the major ontology category in GO (65% of all GO terms). In File S3, we also applied *binary cut* to random GO lists from cellular component and molecular function ontologies. The results were very similar to those obtained with BP terms, where *binary cut* outperformed other clustering methods on semantic similarity matrices.

### General similarity by gene overlap

Many ontologies and gene set collections (*e.g.,* MSigDB gene sets) are only represented as lists of genes for which the similarities are merely measured by gene overlap. We performed a second benchmarking and compared the performance of *binary cut* on four gene overlap measures: Jaccard coefficient, Dice coefficient, overlap coefficient, and kappa coefficient. For this second benchmarking, we again used the 100 random GO lists with 500 BP terms as well as 485 significant GO lists from Expression Atlas datasets.

**Figure 6:**
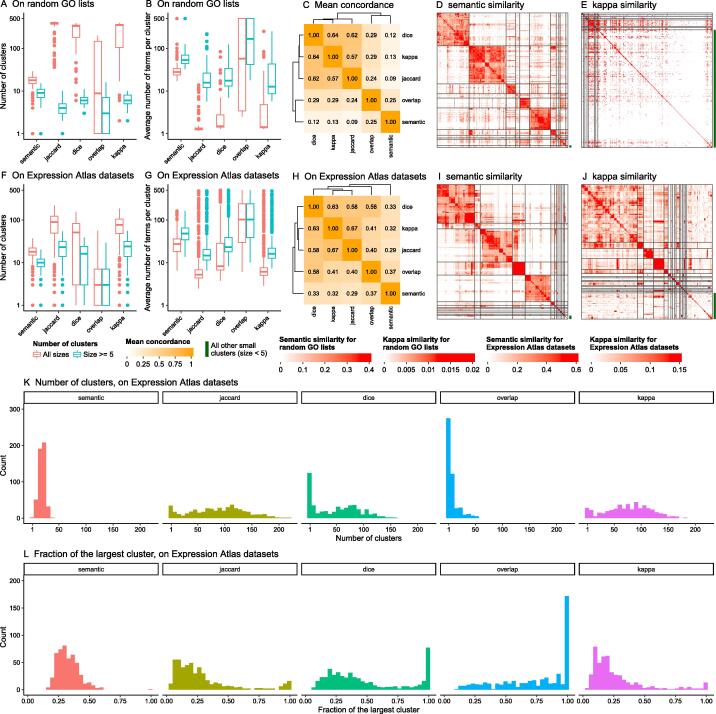
**Comparison of clusterings on similarity matrices by different similarity measures** **A.** Numbers of clusters. **B.** Average numbers of terms per cluster. Y-axes in A and B are on log_10_ scale. **C.** Mean concordance of the clusterings. The definition of concordance can be found in File S13. The analysis was applied to 100 random GO lists with 500 BP terms. **D.** and **E.** Examples of *binary cut* clustering on similarity matrix by semantic and kappa measurement. The two similarity matrices correspond to the same list of random GO terms. **F.**−**J.** Analogous to A−E, but on the functional enrichment results from 485 Expression Atlas datasets. I and J are based on the same Expression Atlas dataset. **K.** Distributions of cluster sizes on similarity matrices from different similarity measures. **L.** Distributions of the fraction of the largest cluster. K and L are based on Expression Atlas datasets.

When GO terms were randomly generated, with similarities measured by Jaccard coefficient, Dice coefficient, and kappa coefficient, 500 terms were split into large numbers of clusters (on average 297 clusters, Figure 6A, red boxes), where on average, less than five terms were in a single cluster (Figure 6B, red boxes). The number of clusters dropped dramatically if only counting clusters with size ≥ 5 (on average 6 clusters for all five similarity measures, Figure 6A, blue boxes). For the clusters with size ≥ 5, the average numbers of GO terms per cluster were 28 with the Jaccard coefficient, 29 with the Dice coefficient, and 31 with the kappa coefficient, while 73 were identified with the semantic similarity measurement (Figure 6B, blue boxes). For the clusters with size < 5, which we defined as “small clusters”, the Jaccard coefficient generated on average 347 small clusters (covering 79.3% of all terms), the Dice coefficient generated 269 small clusters (66.7% of all terms), and the kappa coefficient generated 259 small clusters (65.4% of all terms), while in comparison, semantic similarity only generated 8 small clusters (2.7% of all terms) (see the difference between red and blue boxes in Figure 6A). This implies that *binary cut* generated a huge number of small clusters when based on the similarity matrices of the Jaccard coefficient, Dice coefficient and kappa coefficient, and that these three coefficients could not assign similarities for most of the term pairs, thus they were not efficient for clustering. Examples of the clusterings under different similarity measures can be found in Figure 6D and E and File S9. The overlap coefficient performed differently from the other three gene overlap measures (27.3% agreement of the clusterings, Figure 6C). In 46% of all 100 random GO lists, the 500 GO terms could not even be separated, and the numbers of clusters for them were only identified as one (File S9). The individual heatmaps of overlap similarities showed that in most cases, only one major cluster was generated with a marginal pattern in which a small number of terms showed high similarities to most of the other terms, and no *diagonal block* pattern was observable (File S9); thus *binary cut* had a very weak performance on it. The marginal pattern is due to the definition of the overlap coefficient, according to which the score was normalized by the size of the smaller gene set. In such a setting, a term located in the downstream part of the GO tree, *i.e.*, close to the leaves, has the same overlap coefficients as all its ancestor terms because parent terms include all genes of their child terms. The heatmaps for individual datasets (File S9) showed that, for the randomly sampled GO lists, similarity values calculated by gene overlap were very weak and noisy, which led to the fact that in most cases, terms could not be clustered. In comparison, the semantic similarity matrices generated intermediate numbers of clusters and had clear *diagonal block* patterns (Figure 6D).

Analysis of the Expression Atlas datasets with GO BP ontology also showed that *binary cut* applied to similarity matrices obtained by semantic similarity generated very different clusterings from the four-gene overlap similarity matrices (Figure 6F–J). The cluster sizes obtained from the matrices measured by the Jaccard coefficient, Dice coefficient, and kappa coefficient had very broad ranges where many datasets had more than 100 clusters (on average, 17.1% of all datasets, Figure 6K, an example in Figure 6J). On the other hand, with the Dice coefficient and overlap coefficient, *binary cut* clustered terms into small numbers of clusters for many datasets (less than 5 clusters in 27.2% and 63.9% of all datasets, Figure 6K). In comparison, semantic similarity generated numbers of clusters in a moderate range (11 to 25 for the 10th to the 90th percentile, Figure 6K). Moreover, this analysis showed that the largest cluster comprised more than 80% of all terms in 24.7% of the datasets when using the Dice coefficient and in 54.0% of the datasets when using the overlap coefficient (Figure 6L). This implies that these two coefficients cannot efficiently separate terms in many datasets. Taken together, semantic similarity matrices worked better than gene overlap similarities on real-world datasets.

Besides GO gene sets, we also applied other ontologies and gene set collections to Expression Atlas datasets, *i.e.*, gene sets from DO, KEGG, Reactome, and MSigDB, only with gene overlap as similarity measures (File S10). We found that only the similarity matrices based on gene overlap coefficients of Reactome and MSigDB C4 gene sets can be used for *binary cut* clustering, while the similarity matrices from other gene sets generally showed low consistency between terms and did not have clear *diagonal block* patterns, thus, they are not suited for application of *binary cut*.

### Comparison of enrichment results from multiple lists of genes — a case study

It is a common task to integrate functional enrichment results from multiple lists of genes into one plot, *e.g.*, the enrichment results from up-regulated and down-regulated genes in two-group differential expression analysis. One of the frequently used methods is to make barplot-like graphics where the heights of bars correspond to −log_10_ *P* value or −log_10_ FDR and the enrichment of the up-regulated and down-regulated genes are assigned with positive and negative signs, respectively [33]. This method is limited because users need to pre-select functional terms only to a small number either by selecting top *n* terms with the highest significance or by manually picking representative terms that have distinct biological meanings. The former might lose important terms with less significance, and the latter might be easily affected by subjectivity. Also, this is limited only to two gene lists. Here, we used a public dataset to demonstrate a strategy to efficiently compare enrichment results from multiple gene lists without losing any information, which makes it easy to detect, *e.g.*, the biological functions that are uniquely enriched only in one gene list. It works for arbitrary numbers of gene lists. This strategy was implemented in the function *simplifyGOFromMultipleLists()* in *simplifyEnrichment*.

**Figure 7:**
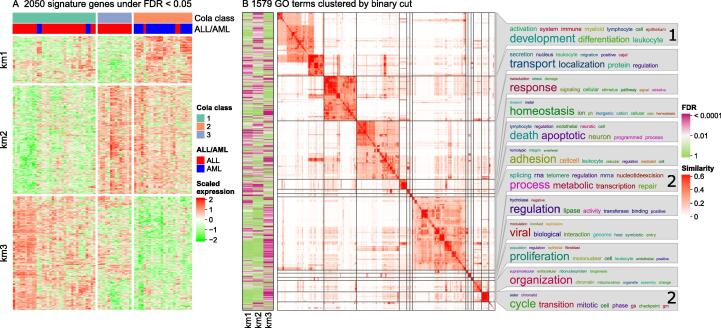
**Compare enrichment results from three gene lists** **A.** Heatmap of the expression of signature genes from a three-group classification of Golub leukemia dataset. The three gene lists were generated by applying *k*-means clustering on rows of the expression matrix. The *z*-score standardization was applied to matrix rows. **B.** GO terms that were significant in any enrichment results of the three gene lists were clustered, and their similarities were visualized as a heatmap. The left heatmap demonstrates whether the GO terms were significant in the corresponding gene list. The labels “1” and “2” on word clouds are explained in the main text.

Figure 7A illustrates a heatmap of expression of signature genes from a three-group classification of the Golub leukemia dataset (golubEsets: exprSets for golub leukemia data; https://www.bioconductor.org/) [34], where rows correspond to genes and columns correspond to samples. The classification of samples was obtained by consensus partitioning with the R package *cola*[35]. The signature genes were additionally clustered into three groups by *k*-means clustering, and the groups were labeled as “km1”, “km2”, and “km3”. GO enrichment analysis was applied to the three groups of genes separately with the R package *clusterProfiler*. To compare the enrichment results from the three gene lists, the *binary cut* was applied directly to the union of the three significant GO term lists (FDR < 0.01), and a heatmap of FDR was put on the left of the GO similarity heatmap to visualize whether the GO terms were significantly enriched in either of the corresponding gene lists (Figure 7B). This strategy keeps all significant GO terms without removing anyone. Also, it allows more specific studies of how the enrichment varies between gene lists. For example, in Figure 7B, it can be observed that genes down-regulated in a subset of acute lymphoblastic leukemia (ALL) samples (km2 group) were more specifically enriched in cell development and differentiation (labeled with “1” in Figure 7B) and genes up-regulated in ALL samples (km3 group) were more specifically enriched in cell cycle and metabolic processes (labeled with “2” in Figure 7B).

This strategy had also been applied in a previous study [35], where it was used to compare the biological functions of gene lists generated by four different feature selection methods for consensus partitioning to assess which feature selection method generates more biologically meaningful features.

## Discussion

Simplifying functional enrichment results is necessary for easy interpretation without missing important biological terms. The essential step to simplify enrichment results is to cluster the functional terms; later downstream analysis can be applied to summarize the major biological functions in each cluster, such as extracting the most significant or representative terms in each cluster [18], [19], [20] or looking for a minimal subset of terms that cover genes from all terms in that cluster [36] (File S11). A proper clustering method should identify groups of terms that provide more distinct separation between clusters and reduce large numbers of terms into an amount easier to read and analyze. In this work, we developed the R package *simplifyEnrichment*, which uses a new method called *binary cut* to efficiently cluster functional terms and summarize the general functions in the obtained clusters by word cloud visualizations.

The *binary cut* is based on the observation that clustering gene sets efficiently is equivalent to the task of identifying diagonal blocks in the similarity matrix of these gene sets after hierarchical clustering of both rows and columns. The algorithm is implemented in two phases: (1) applying divisive clustering and generating a dendrogram on both rows and columns, and (2) cutting the dendrogram and generating clusters. By default, phase (1) relies on the recursive application of PAM with two-group classification, but other partitioning methods, including *k*-means++ and hierarchical clustering with the “ward.D2” method, also performed well.

In the *binary cut* clustering process, a score is calculated based on the two-group partitioning on the current submatrix and is compared against a cutoff to decide whether the corresponding terms are treated as a single cluster or should be further split. In most cases, the default cutoff is robust and works well if a diagonal pattern is observable in the similarity matrix. Nevertheless, there are still cases where manual adjustment on the cutoff needs to be applied, such as a large cluster that is composed of several small and dense subclusters. A typical example is the clustering of the semantic similarity matrices of DO terms (File S3). The cutoff can be fine-tuned according to what levels of similarities users want to keep. *simplifyEnrichment* provides a function *select_cutoff()* which tries a list of cutoffs and compares difference scores, numbers of clusters, and block mean values for the clusterings to decide an optimized cutoff value. In File S12, we demonstrated the use of *select_cutoff()* and how it helps to select a proper cutoff.

Using randomly selected GO terms and a semantic similarity measure, we first compared *binary cut* with 10 different clustering methods. While some methods were generally characterized by over-segmentation (dynamicTreeCut, mclust, and apcluster), others generated intermediate numbers of clusters but either still failed to preserve large clusters with high within-cluster similarities (*kmeans*) or the largest cluster did not show consistent similarities for all term pairs and should be split further (PAM, *hdbscan*, graph community methods). As opposed to that, the *binary cut* was able to simultaneously identify clean small and large clusters. Superior performance was also demonstrated quantitatively using various metrics; in particular, the *binary cut* had the highest difference score, reflecting the most distinct differences in similarity values between within-clusters and between-clusters comparisons.

It is worth noting that when using semantic measures, even for a list of random GO terms, the similarity matrices show clear *diagonal block* patterns. Semantic measures are based on IC; they rely on the IC of the most informative common ancestor of two terms in the GO tree. A GO term has higher IC if it has fewer offspring terms. Thus, in general, the more downstream the ancestor of two terms is in the GO tree, the higher IC the ancestor will provide and the higher similarity the two terms will have. Uniformly sampling the GO terms selects more terms located in the downstream part of the GO tree due to its hierarchical structure; therefore, it is more probable for two terms to reach an ancestor node deeper in the GO tree to contribute a higher IC value, which infers that even random GO lists can have clear *diagonal block* patterns in the benchmark.

The performance of *simplifyEnrichment* was also assessed on actual biological data. To this end, we selected datasets from the Expression Atlas based on the number of significantly differentially expressed genes and the number of significant GO terms after functional enrichment with the GO BP ontology. Again, *binary cut* outperformed other clustering methods. *Binary cut* also outperformed other clustering methods when applied to random GO lists from the cellular component and molecular function ontologies.

As many ontologies and gene set collections (*e.g.*, MSigDB gene sets) do not have DAG structure and are only represented as lists of genes for which the similarities are merely measured by various gene overlap metrics while no semantic similarity measure is available, we performed a second benchmark and compared the performance of *binary cut* when applied to similarity matrices calculated by different similarity measures based on gene overlap, again using both randomly selected GO terms and real data from the Expression Atlas. With the Jaccard coefficient, Dice coefficient, or kappa coefficient as similarity measures, a large number of very small clusters and a small number of bigger clusters were observed on random GO terms, while the overlap coefficient led to massive under-segmentation. As opposed to that, with semantic similarity, these distributions were more equilibrated, intermediate numbers of clusters were generated, and the similarity matrices had clear diagonal block patterns. Also, on the real-world datasets, the semantic similarity measure worked better on GO terms than gene overlap similarity measures, even though the latter showed a more diverse performance. When extending the benchmark to other ontologies and gene set collections, we observed that only gene overlap coefficients of Reactome and MSigDB C4 can be used for clustering with *binary cut*, while DO, KEGG, and other subsets of the MSigDB collections generally showed low consistency between terms and corresponding similarity matrices did not have clear diagonal block patterns. To improve performance on ontologies or gene set collections with no DAG structures, a better definition of term similarity is needed. Current measures based on gene overlap where genes are equally weighted are not good ways to capture consistent correlation structures. The idea is to assign genes with different weights, *e.g.,* weighted by network centralities for pathway ontologies, so that pathways can be assigned high similarity as long as they share key genes in pathway networks.

Finally, we applied *simplifyEnrichment* to a case study in order to demonstrate its ability to integrate functional enrichment results from multiple lists of genes into one plot. The enrichment results of up-regulated and down-regulated genes in a multi-group differential expression analysis were shown in a compact and intuitive manner. Furthermore, results are fed into word cloud visualization, an additional and unique visualization feature of *simplifyEnrichment*. For even deeper exploration and interactive display of enrichment results, *simplifyEnrichment* has in-built functionality to seamlessly launch a Shiny application with the data in the current workspace of the user.

## Conclusion

We described a new clustering algorithm, *binary cut*, for clustering similarity matrices of functional terms. Through comprehensive benchmarks on both simulated and real-world datasets, we demonstrated that *binary cut* could efficiently cluster functional terms where the terms showed more consistent similarities within clusters and were more mutually exclusive between clusters. We implemented the algorithm into the R package *simplifyEnrichment*, which additionally provides functionalities for visualizing, summarizing, and comparing the clusterings. We believe *simplifyEnrichment* will be a useful tool for researchers to rapidly explore their results and obtain key biological messages.

## Code availability

The *simplifyEnrichment* package and the documentation are available at https://bioconductor.org/packages/simplifyEnrichment/ and https://ngdc.cncb.ac.cn/biocode/tools/BT007290. The reports for the analysis of all datasets benchmarked in the paper are available at https://simplifyenrichment.github.io/. The scripts coding the analysis and the versions of R packages for the complete analysis are available at https://github.com/jokergoo/simplifyEnrichment_manuscript. The supplementary files are also available at https://jokergoo.github.io/simplifyEnrichment_supplementary/.

## Competing interests

The authors have declared no competing interests.

## CRediT authorship contribution statement

**Zuguang Gu:** Conceptualization, Methodology, Software, Formal analysis, Validation, Investigation, Writing – original draft, Visualization. **Daniel Hübschmann:** Writing – review & editing, Funding acquisition. Both authors have read and approved the final manuscript.

## Supplementary material

Supplementary File S1 **Definition of gene overlap coefficients**

Supplementary File S2 **Semantic similarity matrices of random GO terms**

Supplementary File S3 **Examples of simplifyEnrichment**

Supplementary File S4 **Examples of five semantic similarity measurements**

Supplementary File S5 **Lower bounds of score *s***

Supplementary File S6 **Interactively visualize the clustering results**

Supplementary File S7 **Global GO term similarity**

Supplementary File S8 **Test partitioning methods**

Supplementary File S9 **Compare similarity measurements – random GO terms**

Supplementary File S10 **Compare similarity measurements – EBI Expression Atlas datasets**

Supplementary File S11 **Compare methods that summarize GO terms in clusters**

Supplementary File S12 **Select cutoff in binary cut clustering**

Supplementary File S13 **Concordance score between two clusterings**
