# Supplementary material for: simplifyEnrichment: A Bioconductor Package for Clustering and Visualizing Functional Enrichment Results
Source: Genomics Proteomics Bioinformatics. 2022 Jun 6;21(1):190–202. doi: 10.1016/j.gpb.2022.04.008 (PMC10373083; doi:10.1016/j.gpb.2022.04.008)
Supplement: Supplementary File S2 — Semantic similarity matrices of random GO terms [file mmc2.zip › supplS02_random_GO_examples.html]

Supplementary file S02. Semantic similarity matrices of random GO terms


# Supplementary file S02. Semantic similarity matrices of random GO terms

#### Zuguang Gu (z.gu@dkfz.de)

#### 2021-11-21

In this supplementary file, we demonstrate the heatmaps of semantic similarities for randomly sampled 500 GO terms, from Biological Process (BP), Molecular Function (MF) and Cellular Component (CC) ontologies. For each ontology category, we show 11 examples. The aim is to demonstrate similarity matrices of random GO terms are very common to show diagonal block patterns.

```
library(simplifyEnrichment)
library(grid)
library(circlize)
library(ComplexHeatmap)
library(GetoptLong)
library(cowplot)

set.seed(123)
col_fun = colorRamp2(c(0, 1), c("white", "red"))
lgd = Legend(title = "Similarity", col_fun = col_fun)
```

## 500 random BP

```
pl = list()
for(i in 1:11) {
    go_id = random_GO(500, "BP")
    mat = GO_similarity(go_id)
    pl[[i]] = grid.grabExpr(draw(Heatmap(mat, col = col_fun, show_row_names = FALSE, show_column_names = FALSE,
        show_row_dend = FALSE, show_column_dend = FALSE, show_heatmap_legend = FALSE, 
        column_title = qq("random BP #@{i}"))))
}
pl[[12]] = lgd@grob
plot_grid(plotlist = pl, nrow = 3)
```

## 500 random MF

```
pl = list()
for(i in 1:11) {
    go_id = random_GO(500, "MF")
    mat = GO_similarity(go_id)
    pl[[i]] = grid.grabExpr(draw(Heatmap(mat, col = col_fun, show_row_names = FALSE, show_column_names = FALSE,
        show_row_dend = FALSE, show_column_dend = FALSE, show_heatmap_legend = FALSE, 
        column_title = qq("random MF #@{i}"))))
}
pl[[12]] = lgd@grob
plot_grid(plotlist = pl, nrow = 3)
```

## 500 random CC

```
pl = list()
for(i in 1:11) {
    go_id = random_GO(500, "CC")
    mat = GO_similarity(go_id)
    pl[[i]] = grid.grabExpr(draw(Heatmap(mat, col = col_fun, show_row_names = FALSE, show_column_names = FALSE,
        show_row_dend = FALSE, show_column_dend = FALSE, show_heatmap_legend = FALSE, 
        column_title = qq("random CC #@{i}"))))
}
pl[[12]] = lgd@grob
plot_grid(plotlist = pl, nrow = 3)
```
