# Supplementary material for: simplifyEnrichment: A Bioconductor Package for Clustering and Visualizing Functional Enrichment Results
Source: Genomics Proteomics Bioinformatics. 2022 Jun 6;21(1):190–202. doi: 10.1016/j.gpb.2022.04.008 (PMC10373083; doi:10.1016/j.gpb.2022.04.008)
Supplement: Supplementary File S3 — Examples of simplifyEnrichment [file mmc3.zip › supplS03_examples.html]

Supplementary file S03. Examples of simplifyEnrichment


# Supplementary file S03. Examples of simplifyEnrichment

This page contains results for functional enrichment clustering done by the **simplifyEnrichment** package.

---

## Random GO

- random\_BP (Based on semantic similarity matrix)
- random\_CC (Based on semantic similarity matrix)
- random\_MF (Based on semantic similarity matrix)
- random\_BP\_dice
- random\_BP\_jaccard
- random\_BP\_kappa
- random\_BP\_overlap

---

## On EBI Expression Atlas datasets

### GO

- EBI\_Expression\_Atlas\_GO\_BP (Based on semantic similarity matrix)
- EBI\_Expression\_Atlas\_GO\_BP\_dice
- EBI\_Expression\_Atlas\_GO\_BP\_jaccard
- EBI\_Expression\_Atlas\_GO\_BP\_kappa
- EBI\_Expression\_Atlas\_GO\_BP\_overlap

### DO

- EBI\_Expression\_Atlas\_DO (Based on semantic similarity matrix)
- EBI\_Expression\_Atlas\_DO\_dice
- EBI\_Expression\_Atlas\_DO\_jaccard
- EBI\_Expression\_Atlas\_DO\_kappa
- EBI\_Expression\_Atlas\_DO\_overlap

### KEGG

- EBI\_Expression\_Atlas\_KEGG\_dice
- EBI\_Expression\_Atlas\_KEGG\_jaccard
- EBI\_Expression\_Atlas\_KEGG\_kappa
- EBI\_Expression\_Atlas\_KEGG\_overlap

### Reactome

- EBI\_Expression\_Atlas\_Reactome\_dice
- EBI\_Expression\_Atlas\_Reactome\_jaccard
- EBI\_Expression\_Atlas\_Reactome\_kappa
- EBI\_Expression\_Atlas\_Reactome\_overlap

### MsigDB\_C2\_CGP

- EBI\_Expression\_Atlas\_MsigDB\_C2\_CGP\_dice
- EBI\_Expression\_Atlas\_MsigDB\_C2\_CGP\_jaccard
- EBI\_Expression\_Atlas\_MsigDB\_C2\_CGP\_kappa
- EBI\_Expression\_Atlas\_MsigDB\_C2\_CGP\_overlap

### MsigDB\_C3\_GTRD

- EBI\_Expression\_Atlas\_MsigDB\_C3\_GTRD\_dice
- EBI\_Expression\_Atlas\_MsigDB\_C3\_GTRD\_jaccard
- EBI\_Expression\_Atlas\_MsigDB\_C3\_GTRD\_kappa
- EBI\_Expression\_Atlas\_MsigDB\_C3\_GTRD\_overlap

### MsigDB\_C3\_MIR\_Legacy

- EBI\_Expression\_Atlas\_MsigDB\_C3\_MIR\_Legacy\_dice
- EBI\_Expression\_Atlas\_MsigDB\_C3\_MIR\_Legacy\_jaccard
- EBI\_Expression\_Atlas\_MsigDB\_C3\_MIR\_Legacy\_kappa
- EBI\_Expression\_Atlas\_MsigDB\_C3\_MIR\_Legacy\_overlap

### MsigDB\_C3\_MIRDB

- EBI\_Expression\_Atlas\_MsigDB\_C3\_MIRDB\_dice
- EBI\_Expression\_Atlas\_MsigDB\_C3\_MIRDB\_jaccard
- EBI\_Expression\_Atlas\_MsigDB\_C3\_MIRDB\_kappa
- EBI\_Expression\_Atlas\_MsigDB\_C3\_MIRDB\_overlap

### MsigDB\_C3\_TFT\_Legacy

- EBI\_Expression\_Atlas\_MsigDB\_C3\_TFT\_Legacy\_dice
- EBI\_Expression\_Atlas\_MsigDB\_C3\_TFT\_Legacy\_jaccard
- EBI\_Expression\_Atlas\_MsigDB\_C3\_TFT\_Legacy\_kappa
- EBI\_Expression\_Atlas\_MsigDB\_C3\_TFT\_Legacy\_overlap

### MsigDB\_C4\_CGN

- EBI\_Expression\_Atlas\_MsigDB\_C4\_CGN\_dice
- EBI\_Expression\_Atlas\_MsigDB\_C4\_CGN\_jaccard
- EBI\_Expression\_Atlas\_MsigDB\_C4\_CGN\_kappa
- EBI\_Expression\_Atlas\_MsigDB\_C4\_CGN\_overlap

### MsigDB\_C4\_CM

- EBI\_Expression\_Atlas\_MsigDB\_C4\_CM\_dice
- EBI\_Expression\_Atlas\_MsigDB\_C4\_CM\_jaccard
- EBI\_Expression\_Atlas\_MsigDB\_C4\_CM\_kappa
- EBI\_Expression\_Atlas\_MsigDB\_C4\_CM\_overlap

### MsigDB\_C7\_IMMUNESIGDB

- EBI\_Expression\_Atlas\_MsigDB\_C7\_IMMUNESIGDB\_dice
- EBI\_Expression\_Atlas\_MsigDB\_C7\_IMMUNESIGDB\_jaccard
- EBI\_Expression\_Atlas\_MsigDB\_C7\_IMMUNESIGDB\_kappa
- EBI\_Expression\_Atlas\_MsigDB\_C7\_IMMUNESIGDB\_overlap
