# Supplementary material for: simplifyEnrichment: A Bioconductor Package for Clustering and Visualizing Functional Enrichment Results
Source: Genomics Proteomics Bioinformatics. 2022 Jun 6;21(1):190–202. doi: 10.1016/j.gpb.2022.04.008 (PMC10373083; doi:10.1016/j.gpb.2022.04.008)
Supplement: Supplementary File S4 — Examples of five semantic similarity measurements [file mmc4.zip › supplS04_examples_of_semantic_measures.html]

 

 

 

 
 
 


 

 

 Supplementary file S04. Examples of five semantic similarity measurements 

 
 
 
 
 
 
 
 
 
 
 

 

 
 


 


 

 

 


 

 


 


 


 Supplementary file S04. Examples of five semantic similarity measurements 
 Zuguang Gu ( z.gu@dkfz.de ) 
 2021-11-21 

 


 
 In this supplementary, we demonstrate the heatmaps of semantic similarity matrices from different semantic measures, which are “Rel”, “Resnik”, “Lin”, “Jiang” and “Wang” (see  the GOSemSim vignette  for more details of the methods). The matrices are calculated based on 500 randomly sampled GO terms, from Biological Process (BP), Molecular Function (MF) and Cellular Component (CC) ontologies. For each ontology category, we show 5 examples. 
 Generally, the similarity matrices generated by the “Rel”, “Resnik” and “Lin” methods show more clear diagonal block patterns. 
 
 500 random BP 
 
 Random 1 
   
 
 
 Random 2 
   
 
 
 Random 3 
   
 
 
 Random 4 
   
 
 
 Random 5 
   
 
 
 
 500 random MF 
 
 Random 1 
   
 
 
 Random 2 
   
 
 
 Random 3 
   
 
 
 Random 4 
   
 
 
 Random 5 
   
 
 
 
 500 random CC 
 
 Random 1 
   
 
 
 Random 2 
   
 
 
 Random 3 
   
 
 
 Random 4 
   
 
 
 Random 5 
   
 
 


 

 

 

 

 


 
 

 
 
