# Supplementary material for: simplifyEnrichment: A Bioconductor Package for Clustering and Visualizing Functional Enrichment Results
Source: Genomics Proteomics Bioinformatics. 2022 Jun 6;21(1):190–202. doi: 10.1016/j.gpb.2022.04.008 (PMC10373083; doi:10.1016/j.gpb.2022.04.008)
Supplement: Supplementary File S5 — Lower bounds of score s [file mmc5.zip › supplS05_lower_bounds_of_score_s.html]

Supplementary file S05. Lower bounds of score s


# Supplementary file S05. Lower bounds of score *s*

#### Zuguang Gu (z.gu@dkfz.de)

#### 2021-11-21

In binary cut algorithm, phase 1, step 1:

> For the similarity matrix \(\mathbf{M}\) of a given gene set, apply a certain partitioning method (e.g., partitioning around medoids (PAM)) with two-group classification on both rows and columns, which partitions \(\mathbf{M}\) into four submatrices, denoted as \(\mathbf{M}\_{11}\), \(\mathbf{M}\_{12}\), \(\mathbf{M}\_{21}\), and \(\mathbf{M}\_{22}\) where the indices represent the groups in the two matrix dimensions. Next calculate the following scores \(s\_{11}\), \(s\_{12}\), \(s\_{21}\) and \(s\_{22}\) for the four submatrices. Taking \(s\_{11}\) as an example, denote \(X\) as a vector of entries in \(\mathbf{M}\_{11}\), \(s\_{11}\) is calculated as \(1-\int\_0^1F\_X(x)dx\) where \(F\_X(x)\) is the cumulative distribution function (CDF) of \(X\). Please note, when calculating \(s\_{11}\) and \(s\_{22}\), entries on the diagonal of \(\mathbf{M}\_{11}\) and \(\mathbf{M}\_{22}\) are excluded. Since the similarity matrix is always symmetric, \(s\_{12}\) and \(s\_{21}\) are equal. \(s\_{11}\) or \(s\_{22}\) is defined to be 1 if \(\mathbf{M}\_{11}\) or \(\mathbf{M}\_{22}\) have only one row. We then define the score \(s\) as \(s=\frac{s\_{11}+s\_{22}}{s\_{11}+s\_{12}+s\_{21}+s\_{22}}\).

A demonstration of \(s\_{11}\), \(s\_{12}\), \(s\_{21}\) and \(s\_{22}\) is in the following figure:

The upper bound of \(s\) is always 1, but the lower bound of \(s\) is not exactly 0.5. In very rare cases, the lower bound of \(s\) is smaller than 0.5, but still very close to 0.5.

First, we demonstrate it with 100 random GO datasets. Here two helper functions are used. `simplifyEnrichment:::cluster_mat()` applies binary cut and it returns a dendrogram object where score \(s\) is stored on every node. Later `dend_node_apply()` is applied to extract \(s\).

```
library(simplifyEnrichment)
set.seed(123)
sl = list()
for(i in 1:100) {
    go_id = random_GO(500)
    mat = GO_similarity(go_id)
    d = simplifyEnrichment:::cluster_mat(mat)
    sl[[i]] = dend_node_apply(d, function(x) attr(x, "score"))
}
```

The scores are visualized by a boxplot and very few scores are less than 0.5:

```
boxplot(sl)
abline(h = 0.5, col = "red", lty = 2)
```

We can quantitatively calculate the fraction of score \(s\) smaller than 0.5:

```
s_small = sapply(sl, function(x) sum(x < 0.5)/length(x))
s_small
```

```
##   [1] 0 0 0 0 0 0 0 0 0 0 0 0 0 0 0 0 0 0 0 0 0 0 0 0 0 0 0 0 0 0 0 0 0 0 0 0 0 0 0 0 0 0 0 0 0 0 0
##  [48] 0 0 0 0 0 0 0 0 0 0 0 0 0 0 0 0 0 0 0 0 0 0 0 0 0 0 0 0 0 0 0 0 0 0 0 0 0 0 0 0 0 0 0 0 0 0 0
##  [95] 0 0 0 0 0 0
```

```
hist(s_small)
```

```
mean(s_small)
```

```
## [1] 0
```

In this random dataset, the probability for \(s\) smaller than 0.5 is zero.

Second, we demonstrate it wit the Expression Atlas dataset. In the following code, `mat_list` contains a list of 468 matrices of GO similarities.

```
mat_list = readRDS("GO_BP_sim.rds")
sl = list()
for(i in seq_along(mat_list)) {
    d = simplifyEnrichment:::cluster_mat(mat_list[[i]])
    sl[[i]] = dend_node_apply(d, function(x) attr(x, "score"))
}
```

Similarly, the probability for \(s\) smaller than 0.5 can be calculated as:

```
s_small = sapply(sl, function(x) sum(x < 0.5)/length(x))
hist(s_small)
```

```
mean(s_small)
```

```
## [1] 0.0003199239
```

The probability is also very tiny.
