# Supplementary material for: simplifyEnrichment: A Bioconductor Package for Clustering and Visualizing Functional Enrichment Results
Source: Genomics Proteomics Bioinformatics. 2022 Jun 6;21(1):190–202. doi: 10.1016/j.gpb.2022.04.008 (PMC10373083; doi:10.1016/j.gpb.2022.04.008)
Supplement: Supplementary File S6 — Interactively visualize the clustering results [file mmc6.zip › supplS06_shiny_app.html]

Supplementary file S06. Interactively visualize the clustering results


# Supplementary file S06. Interactively visualize the clustering results

#### Zuguang Gu (z.gu@dkfz.de)

#### 2021-11-21

The function `export_to_shiny_app()` exports the static similarity heatmap to an interactive Shiny web application. The usage is as follows:

```
library(simplifyEnrichment)
mat = readRDS(system.file("extdata", "random_GO_BP_sim_mat.rds", package = "simplifyEnrichment"))
cl = binary_cut(mat)
export_to_shiny_app(mat, cl)
```

The Shiny app is demonstrated in the following image:
