# Supplementary material for: simplifyEnrichment: A Bioconductor Package for Clustering and Visualizing Functional Enrichment Results
Source: Genomics Proteomics Bioinformatics. 2022 Jun 6;21(1):190–202. doi: 10.1016/j.gpb.2022.04.008 (PMC10373083; doi:10.1016/j.gpb.2022.04.008)
Supplement: Supplementary File S7 — Global GO term similarity [file mmc7.zip › supplS07_global_GO_similarity.html]

Supplementary file S07. Global GO term similarity


# Supplementary file S07. Global GO term similarity

#### Zuguang Gu (z.gu@dkfz.de)

#### 2021-11-21

```
library(simplifyEnrichment)
se_opt$verbose = FALSE
```

In this supplementary, we look at the global GO term similarities for Biological Process (BP) ontology. First we calculate the similarity matrix for all GO BP terms supported in *GOSemSim* package.

```
library(GOSemSim)
semData = godata("org.Hs.eg.db", ont = "BP")
ic = semData@IC

library(simplifyEnrichment)
# This takes very long time
sm = GO_similarity(names(ic), measure = "Rel", ont = "BP")
```

The number of all GO terms in BP ontology:

```
dim(sm)
```

```
## [1] 28641 28641
```

## GO BP terms can be clustered into several major groups

To simplify the similarity matrix, the GO terms which have zero similarity to all other terms are removed.

```
sm_tmp = sm
diag(sm_tmp) = 1
l = rowSums(sm_tmp > 0) > 1
sm = sm[l, l]
rm(sm_tmp)
```

Next we construct the GO tree. The parent-children information is from *GO.db* package. The `GOBPCHILDREN` data object contains lists of direct children terms for every GO terms. If a GO term is a leaf in the GO tree, the value for it is `NA`.

```
library(GO.db)
lt = as.list(GOBPCHILDREN)
lt[1:5]
```

```
## $`GO:0000001`
## [1] NA
## 
## $`GO:0000002`
##      part of          isa 
## "GO:0032042" "GO:0033955" 
## 
## $`GO:0000003`
##          isa          isa      part of          isa          isa          isa 
## "GO:0019953" "GO:0019954" "GO:0022414" "GO:0032504" "GO:0032505" "GO:0075325" 
## 
## $`GO:0000011`
## [1] NA
## 
## $`GO:0000012`
##            regulates negatively regulates positively regulates                  isa 
##         "GO:1903516"         "GO:1903517"         "GO:1903518"         "GO:1903823" 
##                  isa 
##         "GO:1990396"
```

Then we can construct a graph object for the GO tree.

```
df = do.call("rbind", lapply(names(lt), function(nm) {
    data.frame(rep(nm, length(lt[[nm]])), lt[[nm]])
}))
df2 = df[!is.na(df[, 2]), ]

library(igraph)
g = graph.edgelist(as.matrix(df2), directed = TRUE)
g
```

```
## IGRAPH 4ba0288 DN-- 28748 66946 -- 
## + attr: name (v/c)
## + edges from 4ba0288 (vertex names):
##  [1] GO:0000002->GO:0032042 GO:0000002->GO:0033955 GO:0000003->GO:0019953 GO:0000003->GO:0019954
##  [5] GO:0000003->GO:0022414 GO:0000003->GO:0032504 GO:0000003->GO:0032505 GO:0000003->GO:0075325
##  [9] GO:0000012->GO:1903516 GO:0000012->GO:1903517 GO:0000012->GO:1903518 GO:0000012->GO:1903823
## [13] GO:0000012->GO:1990396 GO:0000018->GO:0000019 GO:0000018->GO:0000337 GO:0000018->GO:0010520
## [17] GO:0000018->GO:0010569 GO:0000018->GO:0045191 GO:0000018->GO:0045910 GO:0000018->GO:0045911
## [21] GO:0000018->GO:0061806 GO:0000018->GO:0072695 GO:0000018->GO:1903110 GO:0000019->GO:0032207
## [25] GO:0000019->GO:0045950 GO:0000019->GO:0045951 GO:0000019->GO:1903221 GO:0000022->GO:0032888
## [29] GO:0000022->GO:0051256 GO:0000022->GO:0061804 GO:0000022->GO:0061805 GO:0000022->GO:1902845
## + ... omitted several edges
```

We take the common GO terms in the GO tree and the global similarity matrix.

```
cn = intersect(rownames(sm), unlist(df2))
sm2 = sm[cn, cn]
g = induced_subgraph(g, cn)
sm2 = sm2[V(g)$name, V(g)$name]
```

The final number of GO terms are:

```
dim(sm2)
```

```
## [1] 15988 15988
```

The gobal similarity matrix is clustered by the “louvain” method. Note binary cut is too slow to work on a similarity matrix with 15k GO terms.

```
# This takes around 30 min
cl = cluster_terms(sm2, method = "louvain")
```

The size of clusters:

```
table(cl)
```

```
## cl
##    1    2    3    4 
## 4213 2566 3894 5315
```

To visualize the similarity matrix and the clustering, we only randomly sample 2000 terms.

```
set.seed(123)
ind = sample(length(cl), 2000)
ht_clusters(sm2[ind, ind], cl = cl[ind], exclude_words = c("process", "regulation", "response"))
```

```
## Perform keywords enrichment for 4 GO lists...
```

## GO clusters are dominated by several top GO terms

Next we look at what are the top GO terms in each cluster (*i.e.*, the GO terms located on the top of the GO subtree in each cluster). First we construct subgraph for each cluster.

```
gl = tapply(rownames(sm2), cl, function(go_id) {
    induced_subgraph(g, go_id)
})
```

Since the subgraphs are split from the global tree/graph, they might have several disconnected compoments. We check the sizes of disconnected components in each subgraph.

```
lapply(gl, function(g) {
    table(components(g)$membership)
})
```

```
## $`1`
## 
##    1 
## 4213 
## 
## $`2`
## 
##    1    2 
## 2563    3 
## 
## $`3`
## 
##    1    2 
## 3893    1 
## 
## $`4`
## 
##    1 
## 5315
```

As we can see, in each cluster, there is always a huge components which have majority of the terms while other components have very few terms. Thus, we only take the componets with the largest number of terms.

```
gl2 = lapply(gl, function(g) {
    membership = components(g)$membership
    induced_subgraph(g, membership == which.max(table(membership)))
})
```

Next we extract the top GO terms where the in-degree is zero. And we only want the GO terms which are the parent nodes of a huge GO subtree. The influences a parent node gives to all its descendant nodes is measured by the “spread centrality”.

```
library(CePa)
lapply(gl2, function(g) {
    s1 = degree(g, mode = "in")
    s2 = spread(g, mode = "out")
    s2[s1 == 0]
})
```

```
## $`1`
## GO:0000003 GO:0007610 GO:0032501 GO:0032502 GO:0040007 GO:0044848 GO:0048511 GO:0051704 GO:0110148 
##  92.966667  39.266667 889.378605 955.383874  52.995238   7.166667  18.616667  31.300397   8.583333 
## 
## $`2`
## GO:0006928 GO:0007017 GO:0040011 GO:0051179 GO:0065008 
##   72.63095   22.46190   41.63571  504.32662  170.71158 
## 
## $`3`
## GO:0001775 GO:0002718 GO:0001906 GO:0002376 GO:0007154 GO:0009758 GO:0019740 GO:0022610 GO:0023052 
##  60.685714  17.166667  30.433333 181.271429 291.139286   1.000000   3.416667  37.435714 275.627381 
## GO:0044419 GO:0050896 GO:0065007 GO:0008219 GO:0008283 GO:0019835 
## 100.096429 652.147619 486.051984  85.207143  87.069048   7.333333 
## 
## $`4`
## GO:0008152 GO:0009987 GO:1900673 
##   985.1575  1041.3063     3.0000
```

As shown above, in each cluster, there are some top GO terms that are only the parents of a small number of children terms while some other terms have extremely high spread values which means they are parents of huge number of children terms. We simply remove the top GO terms with spread values less than the mean spread values of all top GO terms.

```
go_list = lapply(gl2, function(g) {
    s1 = degree(g, mode = "in")
    s2 = spread(g, mode = "out")
    go_id = V(g)$name[which(s1 == 0)]
    s = s2[s1 == 0]
    go_id = go_id[s >= mean(s)]
    term = suppressMessages(select(GO.db::GO.db, keys = go_id, columns = "TERM")$TERM)
    paste0(go_id, ": ", term)
})
```

The final major top GO terms in each cluster are:

```
go_list
```

```
## $`1`
## [1] "GO:0032501: multicellular organismal process"
## [2] "GO:0032502: developmental process"           
## 
## $`2`
## [1] "GO:0051179: localization"                    
## [2] "GO:0065008: regulation of biological quality"
## 
## $`3`
## [1] "GO:0002376: immune system process"
## [2] "GO:0007154: cell communication"   
## [3] "GO:0023052: signaling"            
## [4] "GO:0050896: response to stimulus" 
## [5] "GO:0065007: biological regulation"
## 
## $`4`
## [1] "GO:0008152: metabolic process"
## [2] "GO:0009987: cellular process"
```
