# Supplementary material for: simplifyEnrichment: A Bioconductor Package for Clustering and Visualizing Functional Enrichment Results
Source: Genomics Proteomics Bioinformatics. 2022 Jun 6;21(1):190–202. doi: 10.1016/j.gpb.2022.04.008 (PMC10373083; doi:10.1016/j.gpb.2022.04.008)
Supplement: Supplementary File S10 — Compare similarity measurements – EBI Expression Atlas datasets [file mmc10.zip › supplS10_compare_similarity_EBI.html]

Supplementary file S10. Compare similarity measurements - EBI Expression Atlas datasets


# Supplementary file S10. Compare similarity measurements - EBI Expression Atlas datasets

---

We compared the clusterings on the similarity matrices with different similarity measurements.

| dataset | semantic | Jaccard | Dice | overlap | kappa |
| --- | --- | --- | --- | --- | --- |
| Compare similarity measurements - random\_GO\_BP | x | x | x | x | x |
| Compare similarity measurements - EBI\_Expression\_Atlas\_GO\_BP | x | x | x | x | x |
| Compare similarity measurements - EBI\_Expression\_Atlas\_DO | x | x | x | x | x |
| Compare similarity measurements - EBI\_Expression\_Atlas\_KEGG |  | x | x | x | x |
| Compare similarity measurements - EBI\_Expression\_Atlas\_Reactome |  | x | x | x | x |
| Compare similarity measurements - EBI\_Expression\_Atlas\_MsigDB\_C2\_CGP |  | x | x | x | x |
| Compare similarity measurements - EBI\_Expression\_Atlas\_MsigDB\_C3\_GTRD |  | x | x | x | x |
| Compare similarity measurements - EBI\_Expression\_Atlas\_MsigDB\_C3\_MIR\_Legacy |  | x | x | x | x |
| Compare similarity measurements - EBI\_Expression\_Atlas\_MsigDB\_C3\_MIRDB |  | x | x | x | x |
| Compare similarity measurements - EBI\_Expression\_Atlas\_MsigDB\_C3\_TFT\_Legacy |  | x | x | x | x |
| Compare similarity measurements - EBI\_Expression\_Atlas\_MsigDB\_C4\_CGN |  | x | x | x | x |
| Compare similarity measurements - EBI\_Expression\_Atlas\_MsigDB\_C4\_CM |  | x | x | x | x |
| Compare similarity measurements - EBI\_Expression\_Atlas\_MsigDB\_C7\_IMMUNESIGDB |  | x | x | x | x |
