# Supplementary material for: simplifyEnrichment: A Bioconductor Package for Clustering and Visualizing Functional Enrichment Results
Source: Genomics Proteomics Bioinformatics. 2022 Jun 6;21(1):190–202. doi: 10.1016/j.gpb.2022.04.008 (PMC10373083; doi:10.1016/j.gpb.2022.04.008)
Supplement: Supplementary File S11 — Compare methods that summarize GO terms in clusters [file mmc11.zip › supplS11_summarize_clusters.html]

Supplementary file S11. Compare methods that summarize GO terms in clusters


# Supplementary file S11. Compare methods that summarize GO terms in clusters

#### Zuguang Gu (z.gu@dkfz.de)

#### 2021-11-21

In this supplementary, we compare different methods to summarize or represent key functions in GO clusters. The GO list used here corresponds to the significant GO terms for the first group of genes in Figure 7A in the main manuscript.

```
library(cola)
library(ComplexHeatmap)
library(circlize)
library(GetoptLong)
library(simplifyEnrichment)

data(golub_cola)

set.seed(123)

res = golub_cola["ATC:skmeans"]

library(hu6800.db)
mapped_probes = mappedkeys(hu6800ENTREZID)
id_mapping = unlist(as.list(hu6800ENTREZID[mapped_probes]))

lt = functional_enrichment(res, k = 3, id_mapping = id_mapping)
```

```
## - 2050/4116 significant genes are taken from 3-group comparisons
## - on k-means group 1/3, 827 genes
##   - 651/827 (78.7%) genes left after id mapping
##   - gene set enrichment, GO:BP
## - on k-means group 2/3, 359 genes
##   - 317/359 (88.3%) genes left after id mapping
##   - gene set enrichment, GO:BP
## - on k-means group 3/3, 864 genes
##   - 786/864 (91%) genes left after id mapping
##   - gene set enrichment, GO:BP
```

```
df = lt[[1]]
go_id = df[df$p.adjust < 0.01, "ID"]
length(go_id)
```

```
## [1] 618
```

```
mat = GO_similarity(go_id)
cl = cluster_terms(mat)
```

```
## Cluster 618 terms by 'binary_cut'... 23 clusters, used 1.403114 secs.
```

The following four plots are based on the same GO semantic similarity matrix with the same clustering. To summarize each GO term cluster, we use the following four methods:

1. Keywords/word clouds (the first figure). Note in the word cloud annotation, we additionally applied enrichment on keywords and only “significantly overrepresented keywords” are listed in word clouds.
2. Top 3 most significant GO terms in each cluster (the second figure).
3. Top 3 GO terms with the highest IC values in each cluster (the third figure).
4. 2 GO terms whose annotated genes try to be mutually exclusive and the union of annotated genes for the 2 GO terms cover genes in the whole cluster as many as possible (the fourth figure). For each pair of GO terms in a cluster, denote \(A\) as the set of annotated genes for GO term 1, \(B\) as the set of annotated genes for GO term 2, and \(U\) as the set of annotated genes in the whole cluster, a score is assigned to every GO term pair calculated as \(s = (|A \backslash B| + |B \backslash A|)/|U|\) where \(|A|\) is the number of genes in set \(A\) and \(A \backslash B\) is the set of genes in \(A\) but not in \(B\). The GO term pair with the highest score \(s\) is selected as the representative terms in the cluster.

By comparing the following four plots, we can see the word cloud approach, although it only captures the general summaries and visualizes in forms of single words, gives a complete overview of the underlying biological functions. The other three approaches still give highly similar terms in clusters or give too specific terms that can hardly represent the whole cluster.

```
se_opt$verbose = FALSE  # turn off the messages
ht_clusters(mat, cl)
```

```
## Perform keywords enrichment for 10 GO lists...
```

```
align_to = split(1:nrow(mat), cl)
align_to = align_to[sapply(align_to, length) > 5]

selected = lapply(align_to, function(ind) {
    p = df[ind, 2]
    id = df[ind[order(p)[1:3]], 1]
    term = select(GO.db::GO.db, keys = id, columns = "TERM")$TERM
    data.frame(term, c(1, 1, 1))
})

ht_list = ht_clusters(mat, cl, run_draw = FALSE, draw_word_cloud = FALSE) + 
    rowAnnotation(foo = anno_word_cloud(align_to, selected, fontsize_range = c(8, 10)))
draw(ht_list, column_title = "top 3 most significant terms in each cluster")
```

```
selected = lapply(align_to, function(ind) {
    df2 = df[ind, ]
    ic = simplifyEnrichment:::env$semData@IC[df2[, 1]]
    df2 = df2[is.finite(ic), ]
    ic = ic[is.finite(ic)]
    id = df2[order(-ic)[1:3], 1]
    term = select(GO.db::GO.db, keys = id, columns = "TERM")$TERM
    data.frame(term, c(1, 1, 1))
})

ht_list = ht_clusters(mat, cl, run_draw = FALSE, draw_word_cloud = FALSE) + 
    rowAnnotation(foo = anno_word_cloud(align_to, selected, fontsize_range = c(8, 10)))
draw(ht_list, column_title = "top 3 terms with the highest IC in each cluster")
```

```
library(org.Hs.eg.db)
minimal_subset = function(go_id) {
    lt = lapply(go_id, function(x) get(x, org.Hs.egGO2ALLEGS))

    n = length(lt)
    ind_mat = expand.grid(1:n, 1:n)
    ind_mat = ind_mat[ind_mat[, 1] < ind_mat[, 2], , drop = FALSE]
    s = numeric(nrow(ind_mat))

    union = unique(unlist(lt))
    for(i in seq_len(nrow(ind_mat))) {
        s[i] = calc_score(lt[[ind_mat[i, 1]]], lt[[ind_mat[i, 2]]], union)
    }

    go_id[ unlist(ind_mat[order(-s)[1], ]) ]
}

calc_score = function(set1, set2, universe) {
    (length(setdiff(set1, set2)) + length(setdiff(set2, set1)))/length(universe)
}

selected = lapply(align_to, function(ind) {
    id = minimal_subset(df[ind, 1])
    term = select(GO.db::GO.db, keys = id, columns = "TERM")$TERM
    data.frame(term, c(1, 1))
})

ht_list = ht_clusters(mat, cl, run_draw = FALSE, draw_word_cloud = FALSE) + 
    rowAnnotation(foo = anno_word_cloud(align_to, selected, fontsize_range = c(8, 10)))
draw(ht_list, column_title = "top 2 terms with maximal mutual exclusivity on genes in each cluster")
```
