# Supplementary material for: simplifyEnrichment: A Bioconductor Package for Clustering and Visualizing Functional Enrichment Results
Source: Genomics Proteomics Bioinformatics. 2022 Jun 6;21(1):190–202. doi: 10.1016/j.gpb.2022.04.008 (PMC10373083; doi:10.1016/j.gpb.2022.04.008)
Supplement: Supplementary File S13 — Concordance score between two clusterings [file mmc13.zip › supplS13_concordance.html]

Supplementary file S13. Concordance score between two clusterings


# Supplementary file S13. Concordance score between two clusterings

#### Zuguang Gu (z.gu@dkfz.de)

#### 2021-11-21

For the clusterings from two methods, first the cluster labels are adjusted. Denote \(\mathbf{s\_1}\) as the label vector for method 1, \(\mathbf{s\_2}\) as the label vector for method 2, and \(\mathbf{s\_1}\) as the reference labels, we apply `clue::solve_LSAP()` function to generate a mapping function \(m()\) between the two sets of labels to maximize \(\sum^n\_i I(s\_{1i}, m(s\_{2i}))\) where \(n\) is the length of \(\mathbf{s\_1}\) or \(\mathbf{s\_2}\). Denote the adjusted labels for the second method as \(s'\_{2i} = m(s\_{2i})\), the concordance between the two clusterings are calculated as:

\[ \frac{1}{n}\sum\_i^n I(s\_{1i}, s'\_{2i}) \]

where \(I(x, y)\) is an indicator function with value 1 if \(x = y\) and 0 if \(x \ne y\). A concordance of 1 means the two clusterings are identical.
